# A Promising Electrochemical Platform for Dopamine and Uric Acid Detection Based on a Polyaniline/Iron Oxide-Tin Oxide/Reduced Graphene Oxide Ternary Composite

**DOI:** 10.3390/molecules25245869

**Published:** 2020-12-11

**Authors:** Daria Minta, Adam Moyseowicz, Stanisław Gryglewicz, Grażyna Gryglewicz

**Affiliations:** 1 Department of Process Engineering and Technology of Polymer and Carbon Materials, Faculty of Chemistry, Wrocław University of Science and Technology, Gdańska 7/9, 50-344 Wrocław, Poland; daria.minta@pwr.edu.pl (D.M.); adam.moyseowicz@pwr.edu.pl (A.M.); 2 Department of Engineering and Technology of Chemical Processes, Faculty of Chemistry, Wrocław University of Science and Technology, Wybrzeże Wyspiańskiego 42, 50-344 Wrocław, Poland; stanislaw.gryglewicz@pwr.edu.pl

**Keywords:** electrochemical sensor, dopamine and uric acid detection, ternary nanocomposite, hydrothermal treatment

## Abstract

A ternary polyaniline/Fe_2_O_3_-SnO_2_/reduced graphene oxide (PFSG) nanocomposite was prepared using a simple two-step hydrothermal treatment. The composite was applied as a glassy carbon electrode modifier (GCE) to enhance dopamine (DA) and uric acid (UA) detection. The ternary PFSG composite was compared with its binary precursor Fe_2_O_3_-SnO_2_/reduced graphene oxide (FSG). The influence of the modified GCE electrodes on their performance as a sensing platform was determined. GCE/PFSG showed better sensing parameters than GCE/FSG due to the introduction of polyaniline (PANI), increasing the electrocatalytic properties of the electrode towards the detected analytes. GCE/PFSG enabled the detection of low concentrations of DA (0.076 µM) and UA (1.6 µM). The peak potential separation between DA and UA was very good (180 mV). Moreover, the DA oxidation peak was unaffected even if the concentration of UA was ten times higher. The fabricated sensor showed excellent performance in the simultaneous detection with DA and UA limits of detection: LOD_DA_ = 0.15 µM and LOD_UA_ = 6.4 µM, and outstanding long-term stability towards DA and UA, holding 100% and 90% of their initial signals respectively, after one month of use.

## 1. Introduction

In recent years, electrochemical detection has been one of the most widely used techniques for analyte detection, mainly due to its simplicity, fast response and sensitivity [1]. Currently, a large number of studies are being conducted on the electrochemical detection of neurotransmitters such as dopamine and its concomitant analytes [1,2]. Dopamine (DA) plays a significant role in a human organism. Excess DA can increase a person’s susceptibility to depression and drug addiction [3]. In contrast, a DA deficiency is highly related to disorders such as schizophrenia, Parkinson’s disease or HIV infection [4,5]. DA always coexists in human fluids in the presence of different analytes, such as uric acid (UA), ascorbic acid (AA) and epinephrine (EP) [3,6,7]. Among them, UA is crucial for evaluating health [8]. Increased levels of UA can indicate gout or a metabolic syndrome [2,9,10,11]. The concentration of UA in an organism is a hundred times higher than the concentration of DA [12]; from the point of view of electrochemical detection, this large concentration difference can lead to decreased sensitivity towards sensing both analytes and hinder the accuracy of the measurements. In addition, the oxidation potentials of DA and UA are similar, which results in overlapping voltammetric responses and makes selective detection difficult [2,13]. To overcome these difficulties and meet the high demands for electrochemical detection, many studies are being focused on novel electrode materials [2,12]. Among them, binary and ternary nanocomposites based on graphene materials, conductive polymers (CPs) and metal oxides have emerged as a promising sensing platform [12,14,15,16,17].

Graphene-based materials, due to their unique structure, primarily improve the conductivity of composites applied in electrochemical measurement systems [18,19,20]. Owing to their developed active surface area, they provide an increased number of accessible active sites for the detection of analytes [21,22]. The oxygen within the structure of the graphene materials plays a significant role in DA detection. Electroactive oxygen groups increase the affinity of the cationic DA form to the material surface [23,24], however, they decrease the material conductivity. The introduction of CPs is one of the approaches for improving the electrical conductivity of the composite. For this purpose, polyaniline (PANI), polypyrrole (PPy) and poly(3,4-ethylenodioxythiophene) (PEDOT) are widely used in electrochemical applications [25,26,27]. One of the most promising CPs is PANI, due to its simple and cost-effective synthesis and ability to adjust PANI electrochemical properties [25,27,28]. Moreover, CPs are an excellent choice for a sensing platform that facilitates molecule immobilization due to the groups that are present in the polymer structure and are oppositely charged to those that are present in the detected analyte [25].

A wide range of transition metal oxides has also been investigated for use in electrochemical detection systems, including Fe_2_O_3_, SnO_2_, Fe_3_O_4_, ZnO, NiO and Cu_x_O [16,26]. The crucial role of metal oxides in electrochemical detection systems is catalyzing electrochemical reactions [26,29]. The catalytic properties of tin oxide (SnO_2_) have been widely reported, indicating its suitability for sensing applications [30,31,32]. SnO_2_ can also be considered a catalyst support [32,33]. The above-mentioned properties promote the use of SnO_2_ as a carrier of iron oxide (Fe_2_O_3_), which is a low-cost and stable catalyst [31]. Moreover, the as-created mixed metal oxide platform shows a synergistic catalytic effect [31].

Due to their multifunctionality, nanocomposites significantly improve the working parameters of sensors in comparison to their pure counterparts, which has been thoroughly reported recently [26]. To date, most research studies have been mainly focused on the application of binary composites in electrochemical detection methods due to their structural simplicity. Ma et al. [14] reduced a mixture of SnO_2_ nanocrystals and graphene oxide (GO) using AA to obtain reduced graphene oxide (rGO)-SnO_2_ and then applied the as-prepared composite to detect DA. Due to the large number of active sites on the nanocomposite surface, the sensor showed good electrochemical performance, presenting a very low limit of detection (LOD) for DA (6 nM). Despite the low LOD, the proposed sensor worked only in a narrow concentration range (from 0.08 to 30 µM). Another binary graphene-SnO_2_ nanocomposite was proposed by Sun et al. [15]. SnO_2_ nanoparticles, from a hydrothermal synthesis, were mixed with graphene and ultrasonicated. The LOD for DA was 130 nM. In the interference studies, the peak-to-peak separation between DA and UA was 170 mV, indicating no interference of UA with DA. In the detection of UA, a platform based on a hydrothermally synthesized α-Fe_2_O_3_/polyaniline nanotube (PAn NTs) composite was applied [16]. The authors reported that the LOD for UA was 0.038 µM with a linear concentration range of 0.01–5 µM. The normal concentration of UA in human blood ranges from 0.2 to 0.5 mM [12]. Therefore, it can be difficult to detect UA with the above sensor when UA is in excessive concentrations, for instance, in human fluids. Bagheri et al. [17] proposed a ternary Fe_3_O_4_-SnO_2_-Gr nanocomposite as an electrochemical platform for the simultaneous detection of DA, UA and AA. A thermal exfoliation of graphite was used to prepare graphene nanosheets that were mixed with SnO_2_ and Fe_3_O_4_ precursors and sonicated to obtain Fe_3_O_4_-SnO_2_-Gr. The ternary composite exhibited better electrochemical performance in regard to the simultaneous detection of DA, UA and AA than the graphene-modified electrode. The obtained LODs for DA, UA and AA were 7.1, 5.0 and 62 nM, respectively. However, this sensor operated at pH 5.5, which, from the point of view of real sample analysis, requires an additional pH adjustment step. All the aforementioned studies show that multicomponent platforms for DA and UA detection require an appropriate design to overcome issues with sensor sensitivity, selectivity and stability. Binary nanocomposites are very often applied in electrochemical sensing due to their unique properties, but currently, there is a growing interest in using ternary nanocomposites based on CPs, which play a significant role in the detection of various analytes [34].

In this study, we propose a promising platform based on the ternary PANI/Fe_2_O_3_-SnO_2_/rGO nanocomposite (PFSG) for DA and UA detection. We compared ternary nanocomposite performance with its binary precursor Fe_2_O_3_-SnO_2_/rGO (FSG). Despite the complexity of the proposed composites, we applied a simple hydrothermal synthesis route. A beneficial impact on DA detection was observed after the introduction of PANI to the composite structure. The LODs for DA were 1.5 and 0.076 µM for the binary and ternary nanocomposites, respectively. The working pH of the sensors for DA and UA detection was 7.0 and 6.6 respectively, which demonstrates the great applicability of the sensors in real sample analysis due to the pH value being close to the physiological pH of humans. Moreover, the detection of DA was possible even if the concentration of interfering UA was ten times higher. The application of PFSG allowed us to obtain excellent peak-to-peak separation between DA and UA (180 mV). The modified glassy carbon electrode (GCE)/PFSG presents outstanding long-term stability in DA detection, maintaining ~100% of its initial signal after one month of use.

## 2. Results and Discussion

### 2.1. Characterization of Electrode Materials

**Figure 1:**
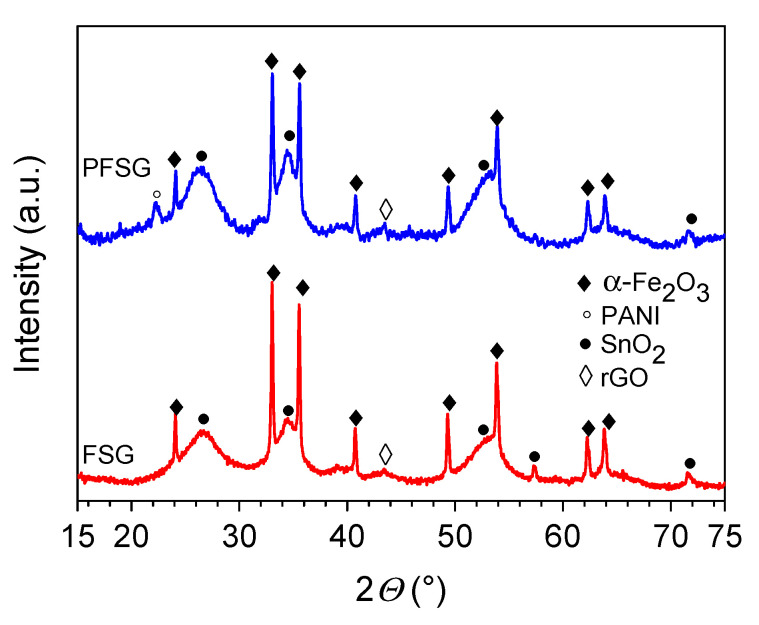
Diffractograms of the FSG and PFSG composites with an annotation of the phases attributed to each peak.

The XRD measurement results of the binary and ternary composites are presented in Figure 1. Under hydrothermal conditions, the resultant mixture of the iron and tin precursors forms two separate crystalline phases. In both the FSG and PFSG composites, visible peaks are attributed to rhombohedral α-Fe_2_O_3_ (JCPDS card no. 33-0664) and tetragonal SnO_2_ (JCPDS card no. 41-1445) [35,36]. The (012) peak of Fe_2_O_3_ at approximately 24° and the SnO_2_ peak at approximately 26.5° (110) overlap with the less intense diffraction peak corresponding to the (002) plane of the rGO graphitic structure [37]. After the introduction of the polymer component, only slight changes in the peaks’ intensity are observed, which indicates that the crystalline structure is preserved in the ternary composite. An additional peak at 22.3° appears in the ternary composite, corresponding to the periodicity parallel to the PANI polymer chains [38].

**Figure 2:**
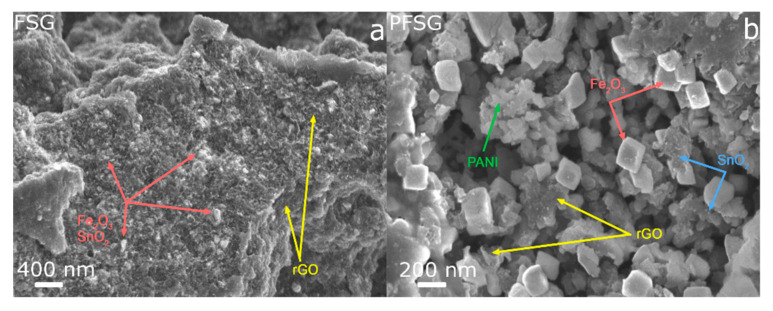
FESEM images of the binary (**a**) and ternary (**b**) composites.

Figure 2 shows the differences in the morphology between the FSG and PFSG composites. FSG presents a characteristic aggregated structure of reduced graphene sheets that are uniformly covered with iron oxide and tin oxide nanoparticles at the surface and within the interconnected rGO structure (Figure 2a). Thus, the Fe_2_O_3_ and SnO_2_ nanoparticles may act as spacers during the hydrothermal reduction of GO, preventing the strong aggregation tendency of graphene sheets [39]. The secondary hydrothermal treatment of the FSG composite in the presence of PANI results in significant changes in the ternary material morphology (Figure 2b). The observable PANI aggregates are present both on the surface and within the pores of the binary precursor, while Fe_2_O_3_ takes on a cubic form with dimensions up to 200 nm in size. PANI partially conceals the iron oxide nanocubes, leading to the formation of the conductive polymer layers on the inorganic component. The smallest nanoparticles on the surface of the rGO are SnO_2_, which maintain their structure compared with their structure in the binary composite.

Nitrogen sorption at 77 K was performed to characterize the porous texture of the investigated composites. Their textural parameters, such as the Brunauer-Emmett-Teller (BET)-specific surface area (S_BET_), the total pore volume (V_t_) and the mesopore volume (V_mes_), are presented in Table 1. According to the IUPAC classification, both FSG and PFSG exhibit a type IV isotherm with an H2 hysteresis loop; however, large adsorption is observed in the wide mesopores of the ternary composite at high p/p_0_ (Supplementary Figure S1a). The BET-specific surface areas of FSG and PFSG are 434 and 206 m^2^ g^−1^, respectively. During the hydrothermal reduction of GO in the presence of the iron and tin precursors, small nanoparticles form on the surface of the graphene nanosheets that prevent their restacking and strong aggregation, which implies a high specific surface area of FSG [39]. The well-developed porous texture of FSG facilitates the insertion of PANI nanoparticles into the material. The proposed hydrothermal approach for the introduction of non-porous CPs into porous materials is extremely beneficial for maintaining the porous structure of the final material, whereas with conventional polymerization, significant blockage of pores usually occurs [37,40,41]. The pore size distribution (PSD) based on the QSDFT (Quenched solid density functional theory) calculations of a ternary composite is close to its binary precursor and presents a mixed micro- and meso-porous character with maxima of approximately 0.9, 1.6 and 3.2 nm (Supplementary Figure S1b). The micropore volume of the PFSG is almost half of the FSG due to the insertion of PANI nanoparticles and subsequent blockage of pores. The sorption analysis results clearly show that after the introduction of significant amounts of non-porous PANI, the composite-specific surface area decreases. However, PFSG still presents a moderately developed porosity, which should provide accessibility to a sufficient amount of active sites for DA and UA adsorption.

**Figure 3:**
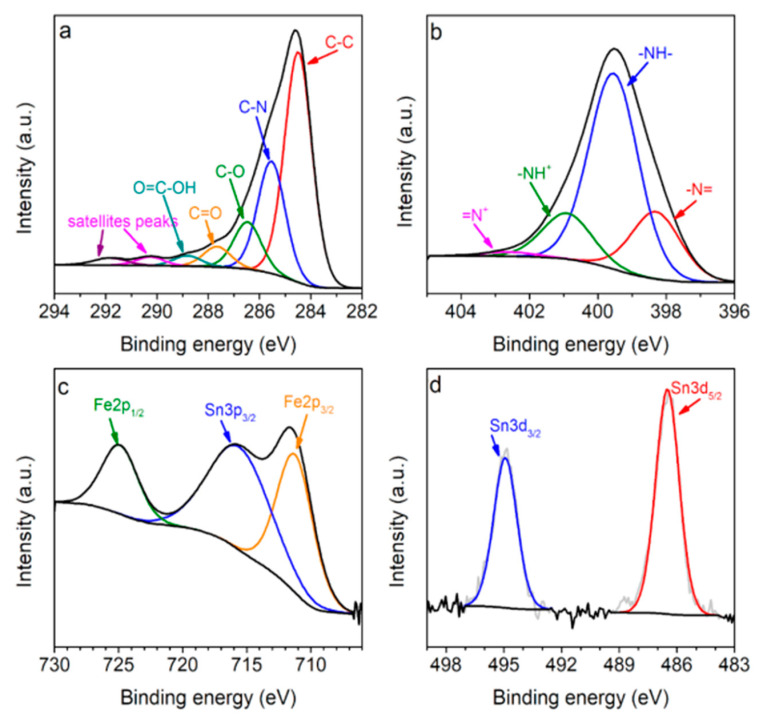
Deconvolutions of (**a**) C1s, (**b**) N1s, (**c**) Fe2p and (**d**) Sn3d for the ternary composite.

The surface chemical characterization of the binary and ternary composites was investigated using the XPS method. The survey spectra of the binary FSG and ternary PFSG composites show the signals from C, N, O, Fe and Sn elements at binding energies of 285, 400, 532, 710 and 485–495 eV, respectively (Supplementary Figure S2). Additionally, the deconvoluted spectra of C1s, N1s, Fe2p and Sn3d of PFSG and FSG are presented in Figure 3 and Supplementary Figure S3, respectively. The C1s high-resolution spectrum of PFSG can be divided into seven components corresponding to C–C bonds (284.5 eV), C–N (285.6 eV), C–O (286.1 eV), carbonyl/quinone (287.5 eV) and carboxyl groups (288.6 eV), and two satellite peaks for the carbon–carbon and carbon–nitrogen bonds at 290 and 291.6 eV, respectively (Figure 3a) [42,43]. The N1s spectra are different for each composite because it originates from ammonia in the case of FSG and from PANI in the case of PFSG (Supplementary Figures S2b and 3b, respectively). The ternary composite N1s spectrum presents four peaks at binding energies of 398.2, 399.6, 400.9 and 402.5 eV, corresponding to imine (–N=), amine (–NH–) and positively charged nitrogen groups of oxidized amine and protonated imine, respectively [44]. In the case of FSG, four peaks are identified and attributed to pyridinic nitrogen (N-6, 398.4 eV), amide and amine nitrogen (NC, 399.5 eV), pyrrolic (N-5, 400.4 eV) and quaternary nitrogen (N-Q, 401.7 eV) [23]. The deconvoluted XPS spectrum of the Fe2p core level of PFSG shows two peaks of Fe2p_1/2_ and Fe2p_3/2_ at binding energies of 724.9 and 711.2 eV, respectively (Figure 3c). The obtained results of the XPS analysis are in good agreement with those reported by Yamashita et al. [45] for α-Fe_2_O_3_. As a final element, the Sn3d deconvolution spectrum presents two distinguishable peaks at 486.5 and 494.9 eV, corresponding to Sn3d_5/2_ and Sn3d_3/2_, respectively. Moreover, the peaks at binding energies of 486.5 and 716 eV in the Fe2p spectrum indicate the presence of SnO_2_ in the PFSG composite [46]. The results from the XPS and XRD measurements confirm the composition of the ternary PFSG composite. Clearly, visible changes in the chemical composition of the ternary composite occurs, leading to an increase in the C and N contents due to the presence of PANI, followed by a decrease in the O and Fe elements associated to the FSG surface being covered with the conductive polymer (Supplementary Figure S2, Table 2).

### 2.2. Electrochemical Performance of Electrodes Towards DA and UA Detection

#### 2.2.1. Characterization of Modified Electrodes

**Figure 4:**
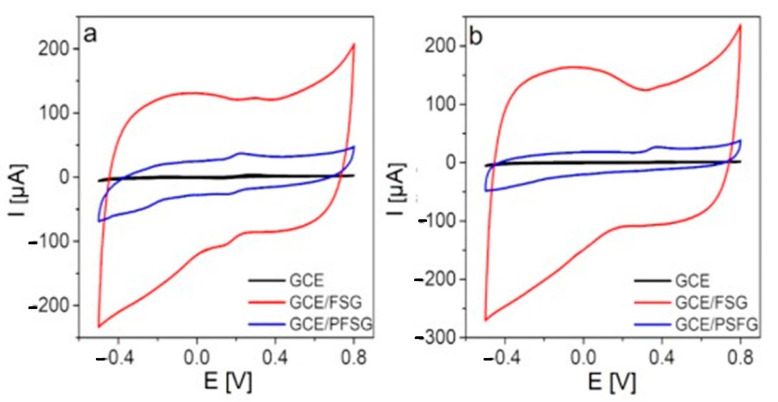
CV curves recorded with the bare GCE, GCE/FSG, GCE/PFSG electrodes in 0.1 M PBS (pH 7.0) containing (**a**) 100 µM DA and (**b**) 300 µM UA.

First, the electrochemical performance of the bare GCE, GCE/FSG and GCE/PFSG electrodes towards DA and UA was evaluated using CV (Figure 4). The bare GCE exhibits a weak signal of the redox reaction of the catechol group in DA to *o*-quinone [12] (282 mV, 3.8 µA). A comparable situation is observed in the application of GCE towards UA detection. The recorded signal of UA oxidation to uric acid imine [47] at a potential of 399 mV is negligible (~1.8 µA). After the GCE is modified with FSG, the oxidation signals of DA and UA are improved to 123.3 µA (288 mV) and 131.9 µA (400 mV), respectively. The application of a ternary nanocomposite (GCE/PFSG) for DA and UA detection enables a significant decrease in the oxidation peak potential for both analytes to 234 mV (45.3 µA) and 378 mV (26.6 µA), respectively. However, the anodic peak currents are lower for GCE/PFSG due to the low contribution of capacitive current, which is undesirable in the detection process and mostly related to the morphology of the material and more developed active surface area of FSG than that of PFSG (434 vs. 206 m^2^ g^−1^) [23]. Moreover, the FSG-modified electrode does not reveal a strong catalytic effect on the oxidation of DA and UA. Thus, the oxidation peak potentials are almost the same as the values recorded on the bare GCE. This result means that the introduction of PANI to the structure of the composite leads to better separation of the FSG sheets, thereby providing more electroactive sites, which is necessary to reduce the activation energies of the reactions [26,48].

#### 2.2.2. Optimization of the PBS pH and Kinetics of DA and UA Oxidation

**Figure 5:**
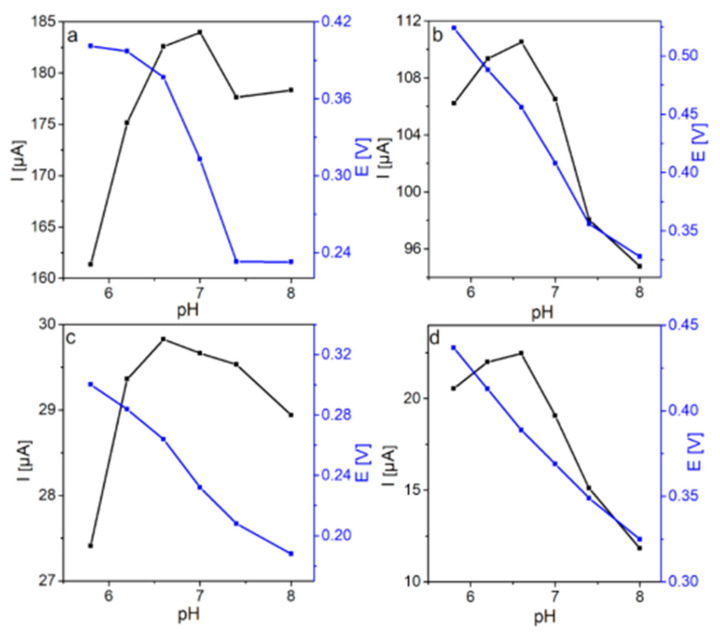
Dependence of anodic peak current and potential on the pH for (**a**) GCE/FSG and (**c**) GCE/PFSG during DA oxidation and (**b**) GCE/FSG and (**d**) GCE/PFSG during UA oxidation.

The influence of the supporting electrolyte pH on DA and UA oxidation was investigated using CV (Supplementary Figure S4). Well-defined peaks of the DA and UA oxidation reactions are observed for both modified electrodes in the pH range of 5.8–8.0. The dependences of the anodic peak potential and peak current on the pH are shown in Figure 5. The highest peak current (184.0 µA) of DA oxidation for the GCE/FSG electrode is observed at pH 7.0, with an anodic peak potential of 312 mV (Figure 5a). A significantly lower anodic peak potential at pH 7.0 (232 mV) than that recorded at pH 6.6 (264 mV) is obtained for the GCE/PFSG electrode in DA detection (Figure 5c). Taking into account the pH value of human fluids, which is close to 7.0 [12], and the comparable values of the peak current recorded with the GCE/PFSG electrode at pH 6.6 and 7.0 (29.82 and 29.66 µA, respectively), the pH value for DA detection in further measurements for both electrodes is established as 7.0. The study on the optimized parameter of the pH solution for UA sensing reveals that the oxidation peak current increases with the pH value to 6.6 and then decreases, resulting in a maximum peak current of 110.52 µA (456 mV) for GCE/FSG and 22.46 µA (388 mV) for GCE/PFSG (Figure 5d). The decrease in the peak current of UA oxidation at pH values higher than 6.6 is probably caused by electrostatic repulsion between the UA molecules, which are deprotonated (pK_a_ = 5.6) under these conditions [2,15], and by the negatively charged electrode surface due to the electronegative oxygen groups present in rGO (Table 2) [12,23,49]. Furthermore, the anodic peak potentials for both electrodes have been shifted to lower values with increasing pH in DA and UA detection, which is in agreement with previously reported works. This result indicates that protons contribute to the DA and UA oxidation processes, which include electron transfer and protonation [12,23]. To determine the kinetics of the reaction mechanism of DA and UA oxidation, the influence of scan rate on the responses from DA and UA with GCE/FSG and GCE/PFSG was investigated. Supplementary Figure S5 shows the CVs recorded at increasing scan rates (2–250 mV s^−1^) on (Figure S5a,b) GCE/FSG and (Figure S5c,d) GCE/PFSG in 0.1 M PBS containing (Figure S5a,c) 100 µM DA and (Figure S5b,d) 300 µM UA. The insets depicted in Supplementary Figure S5 represent the relationship between the anodic peak current and the square root of the scan rate, which is linear for both electrodes, indicating a predominantly diffusion-controlled process for the DA and UA electrode reactions [50,51].

#### 2.2.3. Determination of the Working Parameters of Sensors

**Figure 6:**
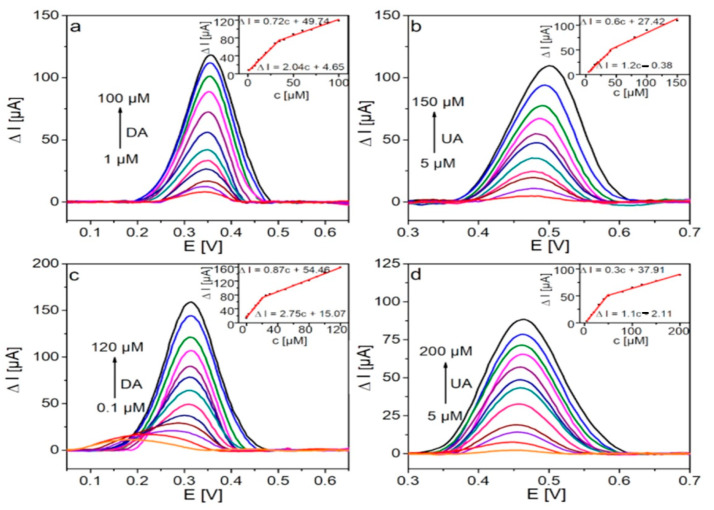
DPV curves recorded with (**a**,**b**) GCE/FSG and (**c**,**d**) GCE/PFSG in the presence of (**a**,**c**) DA in 0.1 M PBS (pH 7.0) and (**b**,**d**) UA in 0.1 M PBS (pH 6.6). Insets show corresponding calibration curves.

The calibration plots of DA and UA oxidation on GCE/FSG and GCE/PFSG were performed by DPV (Differential Pulse Voltammetry), which is a suitable technique to evaluate faradaic processes [52]. Figure 6 shows baseline corrected DPVs for the electrochemical oxidation of DA (Figure 6a,c) and UA (Figure 6b,d) with both modified electrodes. The voltammetric signal increases with increasing concentrations of DA and UA. Insets show the calibration plots of the oxidation peak current vs. the concentration of detected analytes. GCE/FSG and GCE/PFSG operate in two linear concentration ranges. In the low concentration range, the target analyte (DA/UA) reaches the electrode surface faster implying higher current changes. As the concentration of the target analyte increases, the mechanism of adsorption undergoes transition from monolayer adsorption to multilayer adsorption, which results in a slower transport rate [53]. The first linear range (LR) includes low concentrations of DA and UA (GCE/FSG: LR_DA_ = 1–35 µM, LR_UA_ = 5–40 µM; GCE/PFSG: LR_DA_ = 0.1–20 µM, LR_UA_ = 5–50 µM), and the second range includes high analyte concentrations (GCE/FSG: LR_DA_ = 35–100 µM, LR_UA_ = 40–150 µM; GCE/PFSG: LR_DA_ = 20–120 µM, LR_UA_ = 50–300 µM). Both the low and high linear working ranges overlap, which enable the detection of DA and UA over a wide concentration range with high accuracy. The correlation coefficients of the calibration plots were in the range of 0.989–0.997, indicating suitable selection of the linear working scope. The upper limits of the LRs for DA detection with both modified electrodes are comparable. However, a clear difference is observed in the case of detection in the range of low concentrations. The GCE/PFSG electrode enables the detection of DA even at nanomolar concentrations. The lower limit of the LR for GCE/PFSG is ten times lower than that obtained for GCE/FSG (0.1 vs. 1 µM). This outstanding behavior can be explained by the appropriate amount of electronegative oxygen groups in the structure of the ternary composite in comparison to the binary composite [23] and by the improved conductivity of the PFSG material due to the presence of PANI [54]. The role of the electronegative oxygen groups is to attract cationic DA to the surface of the electrode, but these oxygen groups significantly reduce the conductivity of the electrode; therefore, the optimal value of oxygen can improve the working sensor parameters while maintaining good electrical conductivity [23,55]. The introduction of PANI also leads to a significant improvement in the LR of UA detection. The upper limit increases from 150 µM for GCE/FSG to 300 µM for GCE/PFSG. UA at pH 6.6 is in its anionic form, which enables the immobilization of oppositely charged uric acid imines by the positively charged groups of PANI [12,47,49]. The decreased content of negatively charged oxygen groups in PFSG results not only in increased conductivity but also in weakened repulsion between the electrode surface and UA anions [12,49]. Moreover, the protonated amine groups of PANI enhance the attraction of the anionic form of UA. The limits of detection of both electrodes for DA and UA were estimated according to the equation LOD = 3S/b, where *S* is the standard deviation of the blank sample and *b* is the slope of the calibration plot. A great improvement in LOD is observed for the electrode modified with the PFSG nanocomposite in comparison to that of GCE/FSG (0.076 vs. 1.5 µM, respectively). The sensitivity of the sensor is also significantly improved from 2.04 to 2.75 µA µM^−1^ for GCE/FSG and GCE/PFSG, respectively. The better performance of the GCE/PFSG-based sensor can be ascribed to the chemical composition of PFSG, which contains an optimal oxygen content in the composite, and the presence of conductive PANI in the structure of the material, which provides improved electrical conductivity. In the case of UA detection, the LOD value and the sensitivity are comparable for both electrodes: 1.8 µM and 1.2 µA µM^−1^ for GCE/FSG and 1.6 µM and 1.1 µA µM^−1^ for GCE/PFSG, respectively. Moreover, the potential of DA oxidation is reduced from 360 to 320 mV due to the presence of PANI. In the case of UA oxidation, the peak potential shifts towards negative values from 500 to 470 mV for GCE/FSG and GCE/PFSG, respectively. As a result, the DA-UA peak potential separation is 140 and 150 mV for GCE/FSG and GCE/PFSG, respectively. 

The decrease in the oxidation potential of both molecules on GCE/PFSG may result from the separation of FSG sheets by long polymer chains embedded between the composite sheets (Figure 2). This separation enhances the availability of the inorganic part of the composite (Fe_2_O_3_-SnO_2_) for analyte molecules, which plays a catalyzing role in the redox reaction [30,31,32,33]. The performance of the fabricated sensors was compared with that of other binary and ternary nanocomposite-based sensors for DA and UA detection reported in literature (Table 3). The results obtained in this work suggest the great application potential of PFSG ternary nanocomposites for detecting DA, thereby providing a sensing platform with an LOD at a nM concentration and a wide linear range. Fayemi et al. [28] proposed a single analyte sensor that exhibited a lower LOD towards DA (~0.02 µM), but in our work, we demonstrated a double analyte sensor detecting low concentrations of DA (0.076 µM) and UA (1.6 µM).

#### 2.2.4. Selectivity of GCE/PFSG and the Simultaneous Detection of DA and UA

**Figure 7:**
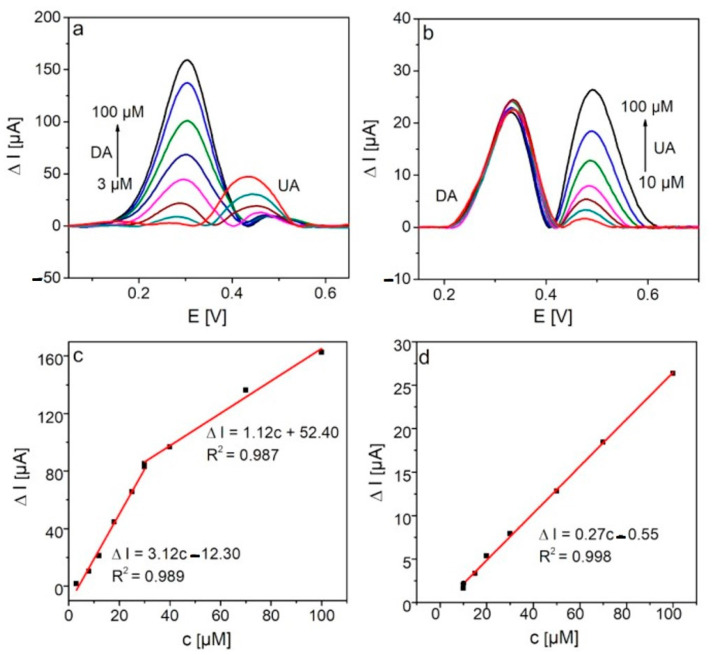
DPV curves recorded on the GCE/PFSG electrode in (**a**) 0.1 M PBS (7.0) containing 100 µM UA and different concentrations of DA and (**b**) 0.1 M PBS (6.6) containing 50 µM DA and different concentrations of UA. Calibration plots of (**c**) DA and (**d**) UA.

Due to the better DA and UA sensing performance of the DA and UA for the GCE/PFSG electrode compared with GCE/FSG, further studies were carried out with an electrochemical detection set and this electrode. To investigate the selectivity of the GCE/PFSG-based sensor towards the detection of DA and UA, DPV measurements were performed when the concentration of one analyte was kept constant (Figure 7). The lowest concentrations of DA and UA that can be detected are 3 and 10 µM respectively, which are higher than the lowest concentrations of analytes detected individually. The peaks corresponding to DA and UA are clearly distinguishable, yielding a peak potential separation of 180 mV, which is significantly higher than that determined during the detection of DA and UA separately (150 mV). With increasing concentrations of DA (Figure 7a), the oxidation peak current of UA decreases, but it does not have a large impact on the applicability of the as-prepared sensor in UA detection. The concentration of UA in human fluids is hundreds of times higher than that of DA [12]. It is worth emphasizing that the peak of DA oxidation is unaffected with increasing concentrations of UA (Figure 7b), indicating the excellent selectivity of the sensor towards DA detection, even if the concentration of UA is much higher than the concentration of DA. Figure 7c,d show the calibration plots corresponding to DA and UA detection, respectively. The sensitivity of the GCE/PFSG towards DA oxidation maintains the same level as that of individual detection (2.75 vs. 3.12 µA µM^−1^). In contrast, a large decrease in the sensitivity, up to 25% of the initial value (1.1 vs. 0.27 µA µM^−1^), is observed in UA detection. This result may indicate that the active sites present on the electrode surface are mainly occupied by DA molecules.

**Figure 8:**
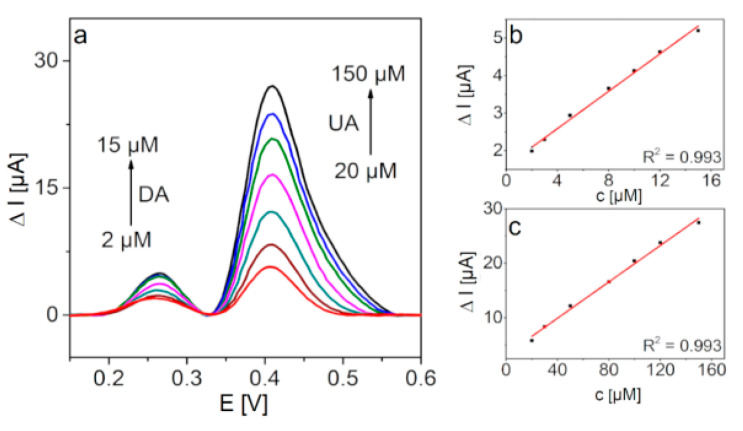
(**a**) DPV curves for the simultaneous detection of DA and UA recorded with GCE/PFSG in 0.1 M PBS (7.0), and (**b**,**c**) show the corresponding calibration plots.

To examine selectivity more accurately, the simultaneous detection of DA and UA was also investigated. Figure 8 shows baseline corrected DPV plots recorded with GCE/PFSG when the concentration of DA is varied from 2 to 15 µM and the UA concentration is kept ten times higher (20–150 µM) and the corresponding calibration plots. The oxidation peaks of both analytes are still clearly distinguishable with a peak potential separation of 160 mV, thus confirming good selectivity of the sensor towards DA and UA detection. The LODs determined in the simultaneous detection experiment are 0.15 and 6.4 µM for DA and UA, respectively. The obtained LODs in the mixture containing DA and UA reveal the possibility of the application of this GCE/PFSG platform in real sample analysis.

#### 2.2.5. Reproducibility, Repeatability and Stability of the GCE/PFSG Sensor towards DA and UA Detection

**Figure 9:**
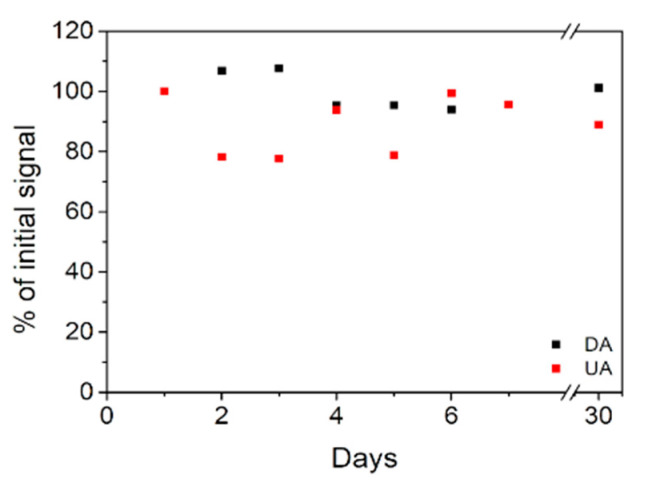
Long-term stability of the GCE/PFSG electrode in DA and UA detection.

The reproducibility of the GCE/PFSG sensor was evaluated by preparing five different modified electrodes and comparing their anodic responses towards DA and UA. The relative standard deviation (RSD) of the DA and UA oxidation signals are 3.2% and 5.1%, respectively. The repeatability was determined by recording the oxidation signal of five independent solutions of 100 µM DA and 300 µM UA. The obtained RSD values are 3.3% and 3.4% for DA and UA, respectively. The low RSD values indicate that the fouling effect at the electrode surface induced by the oxidized forms of DA and UA is minimized [1,19], leading to very good sensor performance. The long-term stability of the modified electrodes was determined by daily measurements of the oxidation peak current for seven days and then taking control measurements after one month (Figure 9). The electrode was immersed in 0.1 M PBS (pH 7.0) while it was not used. After 7 days, the DA and UA oxidation peaks recorded on GCE/PFSG decrease to 95% of the initial signal. After one month, the DA and UA oxidation peak currents maintain approximately 100% and 90% of the initial signal, respectively, indicating the excellent long-term stability of the sensor and its promising application potential. The outstanding long-term stability can be explained by the synergistic effect among the rGO, inorganic Fe_2_O_3_-SnO_2_ phase and PANI. The graphene sheets provide nucleation sites for PANI during the hydrothermal treatment and can be treated as a scaffold for the polymeric chains, which enhances the mechanical strength of the ternary nanocomposite [48,54].

#### 2.2.6. Real Sample Analysis 

To evaluate the suitability of the designed sensor in real sample analysis, standard addition method was applied for three artificial urine samples. The artificial urine was diluted 10 times in 0.1 M PBS, and subsequently certain amounts of DA and UA were added into the sample. The amount of the analyte was estimated by comparing the peak current with the calibration curves (Figure 6). The results are presented in Table 4. The corresponding average recovery values for DA and UA are 104.4% and 85.7% with RSD values of 5.2 and 6.7 respectively, demonstrating a noteworthy potential for the GCE/PFSG electrode for analysis of DA and UA in real samples.

## 3. Materials and Methods

The GO synthesis procedure that uses a modified Hummers method is included in the Supplementary Information.

### 3.1. Preparation of PANI

PANI was prepared using the oxidative polymerization method previously described [40]. Briefly, 500 mg of aniline monomer (Sigma Aldrich, Poznan, Poland) was mixed with 5 mg of surfactant (Triton X-100), 20 mL of Milli-Q water and 10 mL of 1 M HCl. The dispersion was ultrasonicated for 30 min between 0 and 5 °C. Next, ammonium persulfate (APS) as a polymerization initiator was added, and the mixture was stirred in a flask. The polymerization was carried out in the temperature range between 0 and 5 °C for 6 h. The obtained product was filtered and washed with Milli-Q water and methanol until the supernatant reached a neutral pH. Finally, PANI was dried under vacuum at 60 °C for 12 h.

### 3.2. Preparation of FSG

FSG was obtained using a hydrothermal treatment of GO in the presence of Fe(NO_3_)_3_ and SnSO_4_ (Sigma Aldrich, Poznan, Poland). First, 135 mg of aqueous GO solution was mixed with 0.3 mmol Fe(NO_3_)_3_ and 0.15 mmol SnSO_4_ and ultrasonicated for 1 h. Next, 1 mL of 10% *v*/*v* ammonia solution was added to the mixture to establish a pH value of 9. The prepared suspension was then placed in a stainless-steel autoclave (Parr Instrument Company, Moline, IL, USA). The reaction was performed at 180 °C for 8 h under constant stirring at 200 rpm. The resulting product was washed with Milli-Q water and subsequently vacuum-dried overnight at 60 °C. The expected mass ratio of mixed Fe_2_O_3_-SnO_2_ to rGO in the composite was 1:2.

### 3.3. Preparation of PFSG

PFSG was synthesized using an unconventional secondary hydrothermal treatment of polyaniline and the prepared FSG composite. First, 50 mg of FSG was dispersed in Milli-Q water and ultrasonicated for 30 min. Then, 50 mg of polyaniline was added to FSG and ultrasonicated. The mixture was placed into the autoclave, and the reaction was performed at 180 °C for 12 h under constant stirring at 200 rpm. The product was centrifuged three times with Milli-Q water and dried overnight at 60 °C under vacuum. The expected mass ratio among PANI, Fe_2_O_3_-SnO_2_ and rGO in the composite was 50:17:33.

### 3.4. Preparation of Artificial Urine

Artificial urine sample was prepared following the procedure proposed by Chutipongtanate and Thongboonkerd [63]. 2.427 g of urea, 0.034 g of UA, 0.090 g of creatinine, 0.297 g of Na_3_C_6_H_5_O_7_·2H_2_O, 0.634 g of NaCl, 0.450 g of KCl, 0.161 g of NH_4_Cl, 0.089 g of CaCl_2_·2H_2_O, 0.100 g of MgSO_4_·7H_2_O, 0.034 g of NaHCO_3_, 0.003 g of NaC_2_O_4_, 0.258 g of Na_2_SO_4_, 0.100 g of NaH_2_PO_4_·H_2_O and 0.011 g of Na_2_HPO_4_ were dissolved in a 200 mL of Milli-Q water. 

### 3.5. Electrode Modification

The dispersions that were used to modify the GCE were prepared by mixing 4 mg of FSG or PFSG with 1 mL of dimethylformamide (DMF, Sigma Aldrich, Poznan, Poland), Milli-Q water (*v*/*v* of 1:1) and 10 µM of Nafion. Then, the as-prepared dispersions were ultrasonicated for 3 h to obtain a homogenous mixture. Alumina oxide slurries (granulation: 0.3 and 0.05 µm) were used to polish the GCE. After polishing, the electrodes were washed with water. Finally, the cleaned GCEs were modified by drop casting 2.5 µL of the FSG and PFSG dispersions and dried under an infrared lamp.

### 3.6. Materials Characterization and Electrochemical Measurements

The morphology of the prepared composites was examined using field emission scanning electron microscopy (FESEM) with a Merlin Zeiss Instrument (Jena, Germany). The chemical surface composition and distribution of functional groups were analyzed by X-ray photoelectron spectroscopy (XPS) using a PHI 5000 VersaProbe Instrument (Kanagawa, Japan). X-ray diffraction (XRD) patterns were measured using an Ultima IV Rigaku diffractometer with a Cu Kα radiation source (λ = 1.54056 Å). The porous structure of the composites was determined by N_2_ sorption measurements at 77 K using an Autosorb IQ gas sorption analyzer (Quantachrome, Boynton Beach, FL, USA). The quenched solid density functional theory (QSDFT) was used to determine the pore size distribution based on the nitrogen adsorption data.

Electrochemical measurements were performed with a three-electrode cell using a VMP3 potentiostat-galvanostat (Biologic, Grenoble, France). A graphite rod, Ag/AgCl/3.5 M KCl electrode and GCE/FSG or GCE/PFSG electrode were used as the counter, reference and working electrode, respectively. All electrochemical measurements were performed in a phosphate-buffered saline (PBS, 0.1 M) solution at an appropriate pH. Cyclic voltammetry (CV) measurements were performed in a potential range from −0.5 to 0.8 V (vs. Ag/AgCl/3.5 M KCl) at a scan rate (ν_scan_) of 100 mV/s. The calibration plots of DA and UA were obtained by differential pulse voltammetry (DPV) with optimized parameters, including pulse amplitude, pulse width, pulse period and pulse increase (Supplementary Table S1).

## 4. Conclusions

In this paper, we proposed a highly promising polyaniline/Fe_2_O_3_-SnO_2_/rGO nanocomposite platform for DA and UA detection. Using two subsequent hydrothermal treatments, a ternary composite with high PANI content was prepared; additionally, the porous structure of its binary precursor was highly preserved. In comparison to the binary precursor (FSG), the introduction of PANI decreased the oxidation potential of both molecules due to the increased access to catalytic sites in the entire structure of the material, thereby resulting in the good resolution of the DA and UA oxidation peaks. Both fabricated sensors showed very good performance in DA and UA detection. The GCE modification with PFSG provided wider linear concentration ranges for DA and UA detection (0.1–120 µM and 5–300 µM, respectively) in comparison to that of GCE/FSG (1–100 µM, 5–150 µM) and a lower LOD for DA (0.076 µM for GCE/PFSG and 1.5 µM for GCE/FSG). The GCE/FSG and GCE/PFSG sensors enabled the detection of UA with a LOD of 1.8 and 1.6 µM, respectively. The application of GCE/PFSG for simultaneous DA/UA detection in the presence of interfering compounds resulted in good peak separation while maintaining the intensity of the DA oxidation peak, which was almost unaffected. These results clearly indicated the outstanding selectivity of the fabricated sensor towards DA. Moreover, the excellent long-term performance of GCE/PFSG confirmed its potential as a sensing platform, exhibiting 100% of the initial DA signal after 30 days. Additionally, the real sample analysis revealed good applicability of the GCE/PFSG electrode in detection of DA and UA.

## Figures and Tables

**Table 1:** Textural properties of the FSG and PFSG nanocomposites.

Sample	S_BET_	V_t_	V_DR_	V_mes_	V_mes_/V_t_
	(m^2^ g^−1^)	(cm^3^ g^−1^)	(cm^3^ g^−1^)	(cm^3^ g^−1^)	
FSG	434	0.29	0.15	0.14	0.48
PFSG	206	0.21	0.07	0.14	0.67

**Table 2:** Elemental surface compositions of FSG and PFSG.

Sample	C	N	O	Fe	Sn	–N=	–NH–	Doping Level N^+^/N
	(at.%)					(%)		
FSG	63.9	2.6	29.8	3.0	0.9	-	-	-
PFSG	74.6	11.7	12.2	0.8	0.7	21	62	17

**Table 3:** Electrochemical performance of the GCE/FSG and GCE/PFSG electrodes in the detection of DA and UA.

Electrodes	LOD (µM)		Linear Range (µM)		Peak Potential Separation (mV)	Reference
	DA	UA	DA	UA		
GR–SnO_2_/CILE	0.13	-	0.5–500	-	170	[15]
α-Fe_2_O_3_/PAn (NTs)/GCE	-	0.038	-	0.01–5	-	[16]
GCE/PANI-NiO	0.0153	-	2.4–20	-	-	[28]
GCE/PANI-ZnO	0.0166	-	2.4–20	-	-	
GCE/PANI-Fe_2_O_3_	0.0176	-	2.4–20	-	-	
Au-Cu_2_O/rGO	3.9	6.5	10–90	100–900	104	[56]
Au-SiO_2_/GC	1.98	2.58	10–100, 200–500	10–500	215	[57]
HNP-AuAg	0.2	1	5–335	5–425	150	[58]
CdTe QDs-Gr/GC	0.33	0.33	1–600	1–600	-	[59]
PEDOT/Au nanoparticles	0.07	0.08	0.15–330	1.5–150	220	[60]
PtNi@MoS_2_/GCE	0.1	0.1	0.5–250	0.5–1800	150	[61]
MoS_2_ NFs-rGO/ITO	0.12	0.14	5–60	5–60	-	[62]
GCE/FSG	1.5	1.8	1–35, 35–100	5–40, 40–150	140	this work
GCE/PFSG	0.076	1.6	0.1–20, 20–120	5–50, 50–300	150	this work

**Table 4:** Detection results of DA and UA in real sample using GCE/PFSG electrode.

Analyte	Added (µM)	Found (µM)	Recovery (%)	RSD (%)
DA	10	10.5	104.5	5.2
	20	22.2	110.9	
	50	48.8	97.7	
UA	10	9.4	93.5	6.7
	50	41.2	83.9	
	100	79.8	79.8	

## Supplementary Materials

The following are available. Figure S1: Isotherms and pore size distribution for ternary and binary composites, Figure S2: XPS survey spectrum of (a) FSG and (b) PFSG composite, Figure S3: Deconvolutions of (a) C1s, (b) N1s, (c) Fe2p and (d) Sn3d for binary composite, Figure S4: CVs recorded on (a,b) GCE/FSG and (c,d) GCE/PFSG electrodes at different pH values in 0.1 M PBS containing (a,c) 100 µM DA and (b,d) 300 µM of UA, Figure S5: CVs recorded on (a,b) GCE/FSG and (c,d) GCE/PFSG electrodes at different scan rates (2, 5, 10, 20, 50, 75, 100, 150, 200, 250 mV/s) in (a,c) 0.1 PBS (pH 7.0) with 100 µM DA and in (b,d) 0.1 PBS (pH 6.6) with 300 µM UA. Insets show dependence of maximum anodic current against the square root of the scan rate, Table S1: Optimized DPV parameters for the electrodes electrochemical investigation, Table S2: Electrochemical performance of GCE/PFSG in DA and UA detection in the presence of interferences.

## References

[1] Sajid M., Nazal M.K., Mansha M., Alsharaa A., Jillani S.M.S., Basheer C. (2016). Chemically modified electrodes for electrochemical detection of dopamine in the presence of uric acid and ascorbic acid: A review. TrAC Trends Anal. Chem..

[2] Yang Y., Li M., Zhu Z. (2019). A novel electrochemical sensor based on carbon nanotubes array for selective detection of dopamine or uric acid. Talanta.

[3] Arumugasamy S.K., Chellasamy G., Gopi S., Govindaraju S., Yun K. (2020). Current advances in the detection of neurotransmitters by nanomaterials: An update. Trac Trends Anal. Chem..

[4] Hao W., Zhang Y., Fan J., Liu H., Shi Q., Liu W., Peng Q., Zang G. (2019). Copper nanowires modified with graphene oxide nanosheets for simultaneous voltammetric determination of ascorbic acid, dopamine and acetaminophen. Molecules.

[5] Arumugasamy S.K., Govindaraju S., Yun K. (2020). Electrochemical sensor for detecting dopamine using graphene quantum dots incorporated with multiwall carbon nanotubes. Appl. Surf. Sci..

[6] Govindaraju S., Reddy A.S., Kim J., Yun K. (2019). Sensitive detection of epinephrine in human serum via fluorescence enhancement of gold nanoclusters. Appl. Surf. Sci..

[7] Uppachai P., Srijaranai S., Poosittisak S., Isa I.M., Mukdasai S. (2020). Supramolecular electrochemical sensor for dopamine detection based on self-assembled mixed surfactants on gold nanoparticles deposited graphene oxide. Molecules.

[8] Lakshmi D., Whitcombe M.J., Davis F., Sharma P.S., Prasad B.B. (2011). Electrochemical Detection of Uric Acid in Mixed and Clinical Samples: A Review. Electroanalysis.

[9] Wang C., Du J., Wang H., Zou C., Jiang F., Yang P., Du Y. (2014). A facile electrochemical sensor based on reduced graphene oxide and Au nanoplates modified glassy carbon electrode for simultaneous detection of ascorbic acid, dopamine and uric acid. Sens. Actuators B Chem..

[10] Huang W., Cao Y., Chen Y., Zhou Y., Huang Q. (2015). 3-D periodic mesoporous nickel oxide for nonenzymatic uric acid sensors with improved sensitivity. Appl. Surf. Sci..

[11] Yue C.F., Feng P.N., Yao Z.R., Yu X.G., Lin W., Qian Y.M., Guo Y.M., Li L.S., Liu M. (2017). High serum uric acid concentration predicts poor survival in patients with breast cancer. Clin. Chim. Acta.

[12] Feng J., Li Q., Cai J., Yang T., Chen J., Hou X. (2019). Electrochemical detection mechanism of dopamine and uric acid on titanium nitride-reduced graphene oxide composite with and without ascorbic acid. Sens. Actuators B Chem..

[13] Hu B., Liu Y., Wang Z.W., Song Y., Wang M., Zhang Z., Liu C. (2018). Bimetallic-organic framework derived porous Co_3_O_4_/Fe_3_O_4_/C-loaded g-C_3_N_4_ nanocomposites as non-enzymic electrocatalysis oxidization toward ascorbic acid, dopamine acid, and uric acid. Appl. Surf. Sci..

[14] Ma H.F., Chen T.T., Luo Y., Kong F.Y., Fan D.H., Fang H.L., Wang W. (2015). Electrochemical determination of dopamine using octahedral SnO_2_ nanocrystals bound to reduced graphene oxide nanosheets. Microchim. Acta.

[15] Sun W., Wang X., Wang Y., Ju X., Xu L., Li G., Sun Z. (2013). Application of graphene-SnO_2_ nanocomposite modified electrode for the sensitive electrochemical detection of dopamine. Electrochim. Acta.

[16] Mahmoudian M.R., Basirun W.J., Sookhakian M., Woi P.M., Zalnezhad E., Hazarkhani H., Alias Y. (2019). Synthesis and characterization of α-Fe_2_O_3_/polyaniline nanotube composite as electrochemical sensor for uric acid detection. Adv. Powder Technol..

[17] Bagheri H., Pajooheshpour N., Jamali B., Amidi S., Hajian A., Khoshsafar H. (2017). A novel electrochemical platform for sensitive and simultaneous determination of dopamine, uric acid and ascorbic acid based on Fe_3_O_4_-SnO_2_-Gr ternary nanocomposite. Microchem. J..

[18] Bahadir E.B., Sezgintürk M.K. (2016). Applications of graphene in electrochemical sensing and biosensing. TrAC Trends Anal. Chem..

[19] Yang C., Denno M.E., Pyakurel P., Venton B.J. (2015). Recent trends in carbon nanomaterial-based electrochemical sensors for biomolecules: A review. Anal. Chim. Acta.

[20] Chen D., Tang L., Li J. (2010). Graphene-based materials in electrochemistry. Chem. Soc. Rev..

[21] Geim A.K., Novoselov K.S. (2007). The rise of graphene. Nat. Mater..

[22] Huang X., Yin Z., Wu S., Qi X., He Q., Zhang Q., Yan Q., Boey F., Zhang H. (2011). Graphene-based materials: Synthesis, characterization, properties, and applications. Small.

[23] Wiench P., González Z., Menéndez R., Grzyb B., Gryglewicz G. (2018). Beneficial impact of oxygen on the electrochemical performance of dopamine sensors based on N-doped reduced graphene oxides. Sens. Actuators B Chem..

[24] Roy N., Yasmin S., Jeon S. (2020). Effective electrochemical detection of dopamine with highly active molybdenum oxide nanoparticles decorated on 2, 6 diaminopyridine/reduced graphene oxide. Microchem. J..

[25] Naveen M.H., Gurudatt N.G., Shim Y.B. (2017). Applications of conducting polymer composites to electrochemical sensors: A review. Appl. Mater. Today.

[26] Dakshayini B.S., Reddy K.R., Mishra A., Shetti N.P., Malode S.J., Basu S., Naveen S., Raghu A.V. (2019). Role of conducting polymer and metal oxide-based hybrids for applications in ampereometric sensors and biosensors. Microchem. J..

[27] Long Y.Z., Li M.M., Gu C., Wan M., Duvail J.L., Liu Z., Fan Z. (2011). Recent advances in synthesis, physical properties and applications of conducting polymer nanotubes and nanofibers. Prog. Polym. Sci..

[28] Fayemi O.E., Adekunle A.S., Kumara Swamy B.E., Ebenso E.E. (2018). Electrochemical sensor for the detection of dopamine in real samples using polyaniline/NiO, ZnO, and Fe_3_O_4_ nanocomposites on glassy carbon electrode. J. Electroanal. Chem..

[29] Bharath G., Alhseinat E., Madhu R., Mugo S.M., Alwasel S., Harrath A.H. (2018). Facile synthesis of Au@α-Fe_2_O_3_@RGO ternary nanocomposites for enhanced electrochemical sensing of caffeic acid toward biomedical applications. J. Alloys Compd..

[30] Kumar A., Rout L., Dhaka R.S., Samal S.L., Dash P. (2015). Design of a graphene oxide-SnO_2_ nanocomposite with superior catalytic efficiency for the synthesis of β-enaminones and β-enaminoesters. Rsc Adv..

[31] Wang N., Du Y., Ma W., Xu P., Han X. (2017). Rational design and synthesis of SnO_2_-encapsulated *α*-Fe_2_O_3_ nanocubes as a robust and stable photo-Fenton catalyst. Appl. Catal. B Environ..

[32] Rumyantseva M., Kovalenko V., Gaskov A., Makshina E., Yuschenko V., Ivanova I., Ponzoni A., Faglia G., Comini E. (2006). Nanocomposites SnO_2_/Fe_2_O_3_: Sensor and catalytic properties. Sens. Actuators B Chem..

[33] Wang Y., Hu X., Zheng K., Wei X., Zhao Y. (2018). Effect of SnO_2_ on the structure and catalytic performance of Co_3_O_4_ for N_2_O decomposition. Catal. Commun..

[34] Radhakrishnan S., Krishnamoorthy K., Sekar C., Wilson J., Kim S.J. (2015). A promising electrochemical sensing platform based on ternary composite of polyaniline–Fe_2_O_3_–reduced graphene oxide for sensitive hydroquinone determination. Chem. Eng. J..

[35] Lassoued A., Dkhil B., Gadri A., Ammar S. (2017). Control of the shape and size of iron oxide (α-Fe_2_O_3_) nanoparticles synthesized through the chemical precipitation method. Results Phys..

[36] Lin J.Y., Chou M.H., Kuo Y.C. (2014). Rapid synthesis of tin oxide decorated carbon nanotube nanocomposities as anode materials for lithium-ion batteries. J. Alloys Compd..

[37] Moyseowicz A., Śliwak A., Miniach E., Gryglewicz G. (2017). Polypyrrole/iron oxide/reduced graphene oxide ternary composite as a binderless electrode material with high cyclic stability for supercapacitors. Compos. Part B Eng..

[38] Neelgund G.M., Oki A. (2011). A facile method for the synthesis of polyaniline nanospheres and the effect of doping on their electrical conductivity. Polym. Int..

[39] Li J., Östling M. (2013). Prevention of graphene restacking for performance boost of supercapacitors—A review. Crystals.

[40] Moyseowicz A., Gryglewicz G. (2019). Hydrothermal-assisted synthesis of a porous polyaniline/reduced graphene oxide composite as a high-performance electrode material for supercapacitors. Compos. Part B Eng..

[41] Moyseowicz A., Pajak K., Gajewska K., Gryglewicz G. (2020). Synthesis of polypyrrole/reduced graphene oxide hybrids via hydrothermal treatment for energy storage applications. Materials.

[42] Moyseowicz A., González Z., Menéndez R., Gryglewicz G. (2018). Three-dimensional poly(aniline-co-pyrrole)/thermally reduced graphene oxide composite as a binder-free electrode for high-performance supercapacitors. Compos. Part B Eng..

[43] Golczak S., Kanciurzewska A., Fahlman M., Langer K., Langer J.J. (2008). Comparative XPS surface study of polyaniline thin films. Solid State Ions.

[44] Rajagopalan R., Iroh J.O. (2003). Characterization of polyaniline-polypyrrole composite coatings on low carbon steel: A XPS and infrared spectroscopy study. Appl. Surf. Sci..

[45] Yamashita T., Hayes P. (2008). Analysis of XPS spectra of Fe 2+ and Fe 3+ ions in oxide materials. Appl. Surf. Sci..

[46] Lee K., Shin S., Degen T., Lee W., Yoon Y.S. (2017). In situ analysis of SnO_2_/Fe_2_O_3_/RGO to unravel the structural collapse mechanism and enhanced electrical conductivity for lithium-ion batteries. Nano Energy.

[47] Roy P.R., Okajima T., Ohsaka T. (2004). Simultaneous electrochemical detection of uric acid and ascorbic acid at a poly(*N*,*N*-dimethylaniline) film-coated GC electrode. J. Electroanal. Chem..

[48] Zhang J., Zhao X.S. (2012). Conducting polymers directly coated on reduced graphene oxide sheets as high-performance supercapacitor electrodes. J. Phys. Chem. C.

[49] Sheng Z.H., Zheng X.Q., Xu J.Y., Bao W.J., Wang F.B., Xia X.H. (2012). Electrochemical sensor based on nitrogen doped graphene: Simultaneous determination of ascorbic acid, dopamine and uric acid. Biosens. Bioelectron..

[50] Sundar S., Venkatachalam G., Kwon S.J. (2018). Biosynthesis of copper oxide (Cuo) nanowires and their use for the electrochemical sensing of dopamine. Nanomaterials.

[51] Zhang X., Zheng J. (2020). High-index {hk0} facets platinum concave nanocubes loaded on multiwall carbon nanotubes and graphene oxide nanocomposite for highly sensitive simultaneous detection of dopamine and uric acid. Talanta.

[52] Ozkan S.A., Kauffmann J.-M., Zuman P. (2015). Electroanalysis in Pharmaceutical Biomedical and Pharmaceutical Sciences.

[53] Zhuang Z., Li J., Xu R., Xiao D. (2011). Electrochemical detection of dopamine in the presence of ascorbic acid using overoxidized polypyrrole/graphene modified electrodes. Int. J. Electrochem. Sci..

[54] Wang H., Lin J., Shen Z.X. (2016). Polyaniline (PANi) based electrode materials for energy storage and conversion. J. Sci. Adv. Mater. Devices.

[55] Rahman M.M., Lee J.J. (2019). Electrochemical dopamine sensors based on graphene. J. Electrochem. Sci. Technol..

[56] Aparna T.K., Sivasubramanian R., Dar M.A. (2018). One-pot synthesis of Au-Cu_2_O/rGO nanocomposite based electrochemical sensor for selective and simultaneous detection of dopamine and uric acid. J. Alloys Compd..

[57] Immanuel S., Aparna T.K., Sivasubramanian R. (2019). A facile preparation of Au-SiO_2_ nanocomposite for simultaneous electrochemical detection of dopamine and uric acid. Surf. Interfaces.

[58] Hou J., Xu C., Zhao D., Zhou J. (2016). Facile fabrication of hierarchical nanoporous AuAg alloy and its highly sensitive detection towards dopamine and uric acid. Sens. Actuators B Chem..

[59] Yu H., Jiang J., Zhang Z., Wan G., Liu Z., Chang D., Pan H. (2017). Preparation of quantum dots CdTe decorated graphene composite for sensitive detection of uric acid and dopamine. Anal. Biochem..

[60] Ali A., Jamal R., Abdiryim T., Huang X. (2017). Synthesis of monodispersed PEDOT/Au hollow nanospheres and its application for electrochemical determination of dopamine and uric acid. J. Electroanal. Chem..

[61] Ma L., Zhang Q., Wu C., Zhang Y., Zeng L. (2019). PtNi bimetallic nanoparticles loaded MoS_2_ nanosheets: Preparation and electrochemical sensing application for the detection of dopamine and uric acid. Anal. Chim. Acta.

[62] Guo X., Yue H., Song S., Huang S., Gao X., Chen H., Wu P., Zhang T., Wang Z. (2020). Simultaneous electrochemical determination of dopamine and uric acid based on MoS_2_ nanoflowers-graphene/ITO electrode. Microchem. J..

[63] Chutipongtanate S., Thongboonkerd V. (2010). Systematic comparisons of artificial urine formulas for in vitro cellular study. Anal. Biochem..

