# Supplementary material for: A Promising Electrochemical Platform for Dopamine and Uric Acid Detection Based on a Polyaniline/Iron Oxide-Tin Oxide/Reduced Graphene Oxide Ternary Composite
Source: Molecules. 2020 Dec 11;25(24):5869. doi: 10.3390/molecules25245869 (PMC7763624; doi:10.3390/molecules25245869)
Supplement: Supplementary file 1 [file molecules-25-05869-s001.pdf]

Supplementary material

A promising electrochemical platform for dopamine and uric acid detection based on a polyaniline/iron oxide-tin oxide/reduced graphene oxide ternary composite

Daria Minta [1], Adam Moyseowicz [1], Stanisław Gryglewicz [2], Grażyna Gryglewicz [1,*]

[1] Department of Process Engineering and Technology of Polymer and Carbon Materials, Faculty of Chemistry, Wrocław University of Science and Technology, Gdańska 7/9, 50-344 Wrocław, Poland; daria.minta@pwr.edu.pl (D.M.); adam.moyseowicz@pwr.edu.pl (A.M)

[2] Department of Engineering and Technology of Chemical Processes, Faculty of Chemistry, Wrocław University of Science and Technology, Wybrzeże Wyspiańskiego 42, 50-344 Wrocław, Poland; stanislaw.gryglewicz@pwr.edu.pl (S.G.)

* Correspondence: grazyna.gryglewicz@pwr.edu.pl; Tel.: +48-71-320-6398; Fax: +48-71-320-6506 (G.G.)

Preparation of graphene oxide

The synthesis of GO was performed using modified Hummers method which was described previously [33]. In brief, in a flask, 2 g of graphite (C-NERGY KS 6L, TimCal) was mixed with 96 mL of H_2SO_4 (98 %) and 2g of $NaNO_3$ (Sigma Aldrich). Next, the reaction mixture was cooled down to 7-9 °C in an ice bath and 12 g of $KMnO_4$ (Sigma Aldrich) was added under constant stirring. Then the mixture was heated to 35 °C and stirred for 3 hours. After that, 400 mL of H_2O_2 (3 wt.%, Sigma Aldrich) was introduced to the flask. Finally, the solid product was washed with Milli-Q water until the supernatant reached a neutral pH. The resulting graphite oxide (GrO) was treated with 5 wt.% HCl solution (Sigma Aldrich) and then washed with Milli-Q water. Finally, GrO was exfoliated in a sonication bath for 2 hours to obtain a GO aqueous suspension.

Table S1. Optimized DPV parameters for the electrodes electrochemical investigation

Electrodes	t [s]		P_H [mV]		P_W [ms]		S_H [mV]		S_T [ms]	
	DA	UA	DA	UA	DA	UA	DA	UA	DA	UA
GCE/FSG	20		175		25		5		100	50
GCE/PFSG									50	30

t – holding time at starting potential

P_H – pulse amplitude

P_W – pulse width

S_H – pulse increment

S_T – pulse period

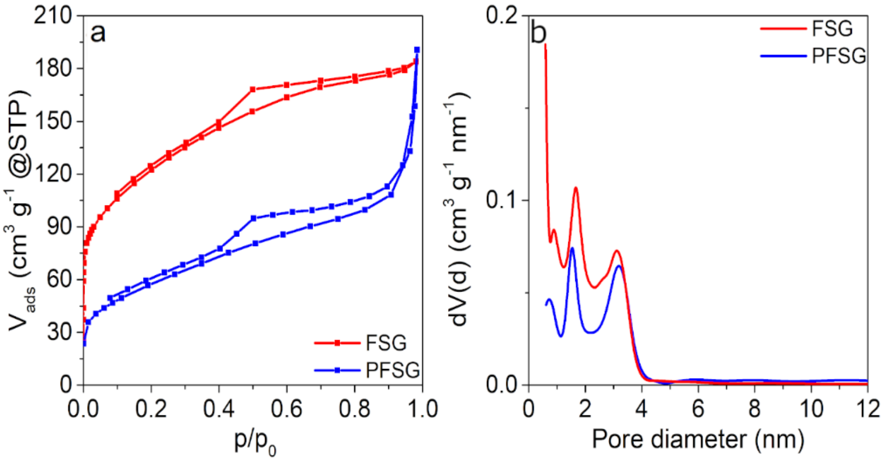

Figure S1. Isotherms and pore size distribution for ternary and binary composites.

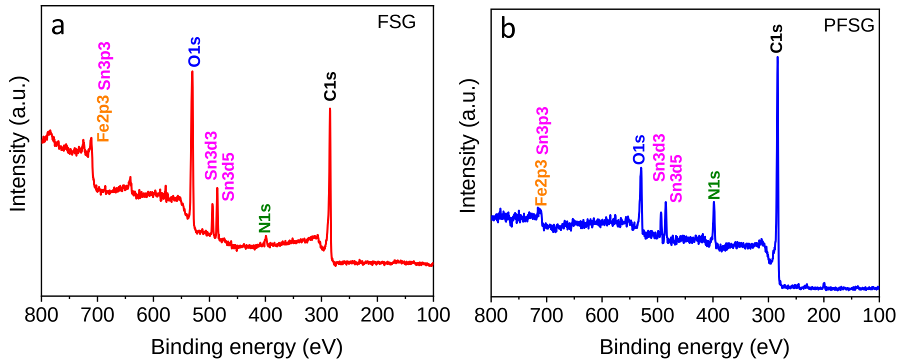

Figure S2. XPS survey spectrum of (a) FSG and (b) PFSG composite.

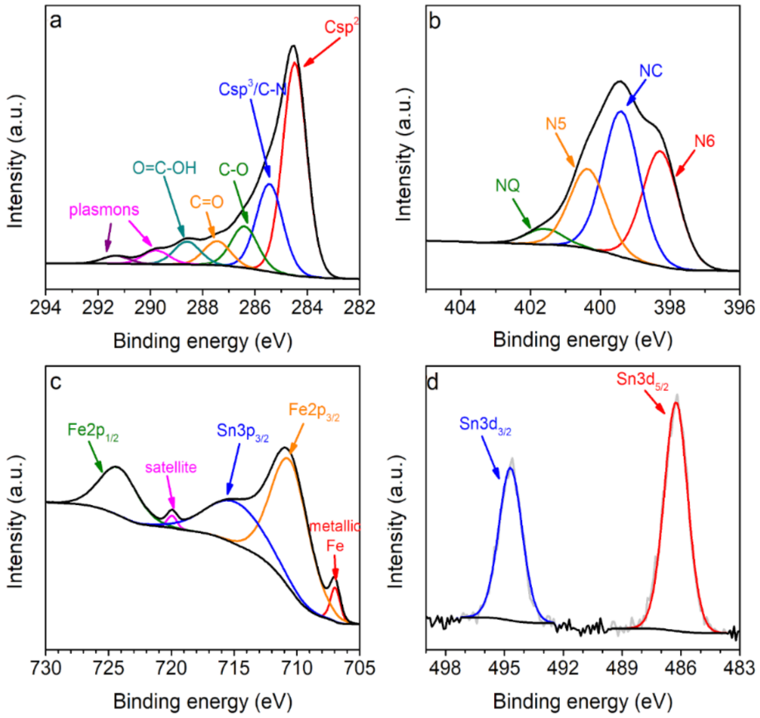

Figure S3. Deconvolutions of (a) C1s, (b) N1s, (c) Fe2p and (d) Sn3d for binary composite.

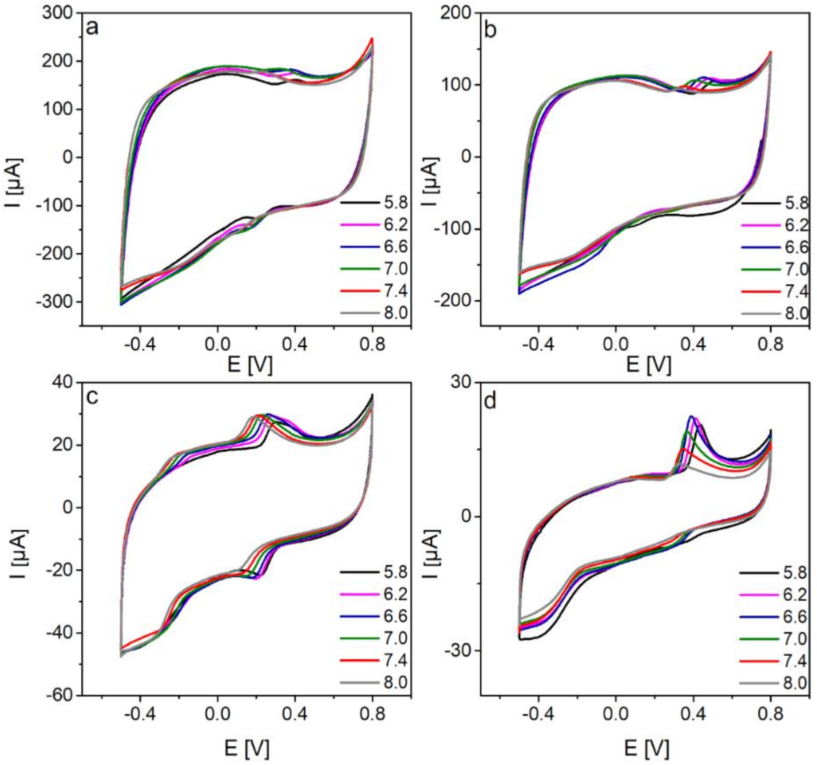

Figure S4. CVs recorded on (a,b) GCE/FSG and (c,d) GCE/PFSG electrodes at different pH values in 0.1 M PBS containing (a,c) 100 µM DA and (b,d) 300 µM of UA.

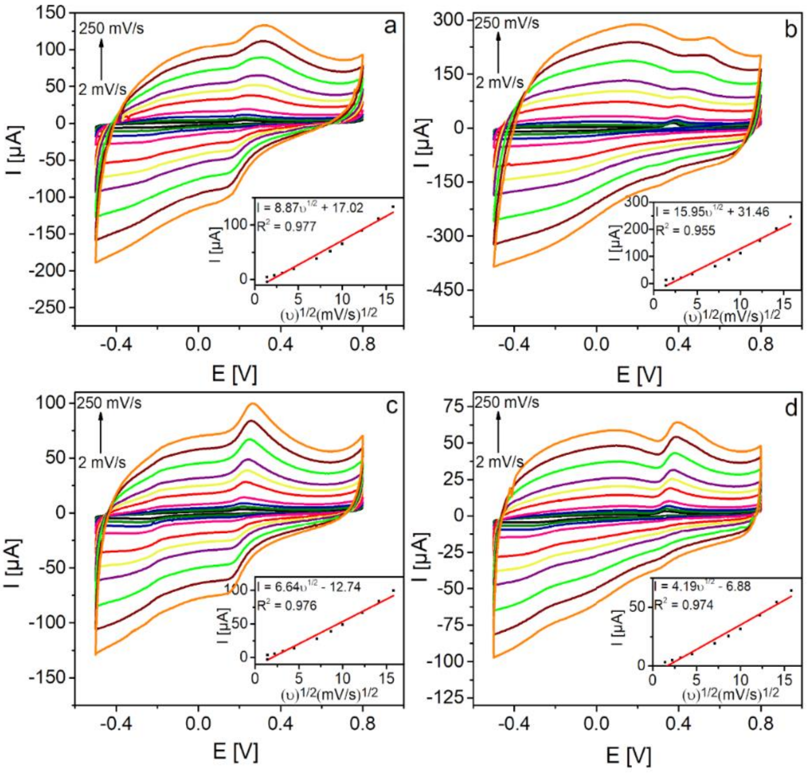

Figure S5. CVs recorded on (a,b) GCE/FSG and (c,d) GCE/PFSG electrodes at different scan rates (2, 5, 10, 20, 50, 75, 100, 150, 200, 250 mV/s) in (a,c) 0.1 PBS (pH 7.0) with 100 μM DA and in (b,d) 0.1 PBS (pH 6.6) with 300 μM UA. Insets show dependence of maximum anodic current against the square root of the scan rate.

Table S2. Electrochemical performance of GCE/PFSG in DA and UA detection in the presence of interferences.

Electrodes	LOD [μM]		Linear range [μM]		Sensitivity [μA μM^{-1}]	
	DA	UA	DA	UA	DA	UA
GCE/PFSG	1.7	2.5	3 – 30, 30 – 100	10 – 100	3.2, 1.1	0.3
